# Pathogenic role of glycan-specific IgG antibodies in IgA nephropathy

**DOI:** 10.1186/s12882-017-0722-3

**Published:** 2017-09-29

**Authors:** Yan-feng Zhao, Li Zhu, Li-jun Liu, Su-fang Shi, Ji-cheng Lv, Hong Zhang

**Affiliations:** 1 Renal Division, Department of Medicine, Peking University First Hospital, Beijing, China; 2 Peking University Institute of Nephrology, Beijing, China; 3 Key Laboratory of Renal Disease, Ministry of Health of China, Beijing, China; 4 Key Laboratory of Chronic Kidney Disease Prevention and Treatment (Peking University), Ministry of Education, Beijing, China; 5 Renal Division, Department of Medicine, Peking University First Hospital, Peking University Institute of Nephrology, No 8, Xishiku Street, Xicheng District, Beijing, 100034 China

**Keywords:** IgA nephropathy, Glycan-specific IgG antibody, Galactose-deficient IgA1

## Abstract

**Background:**

Accumulating evidences proved the important roles of circulating IgA1-containing immune complexes (cIgA1) in IgA nephropathy (IgAN). Galactose-deficient IgA1 (Gd-IgA1) and glycan-specific IgG antibody have been identified as major components in cIgA1. Before, Gd-IgA1 was reported as a vital factor in IgAN, partly via of its pathogenic role to induce mesangial cells activation. However, we still lack direct evidences to clarify the biological effect of glycan-specific IgG antibody in IgAN.

**Methods:**

In the present study, we enrolled 35 IgAN patients and 17 age- and sex-matched healthy controls. Using uniform aberrant glycosylated IgA1 molecules, and IgG from different individuals, we in vitro prepared IgG-ddIgA1 complexes, and compared the biological differences among these immune complexes regarding their proliferative and inflammatory effects on mesangial cells.

**Results:**

IgG-ddIgA1 complexes from both patients with IgA nephropathy (IgAN-IgG-dd-IgA1) and healthy controls (HC-IgG-dd-IgA1) could induce the proliferation of mesangial cells and up-regulate expression of MCP-1, IL-6 and CXCL1. The levels of mesangial cells proliferation induced by IgAN-IgG-dd-IgA1 were significantly higher than those induced by HC-IgG-dd-IgA1 (1.10 ± 0.05 vs. 1.03 ± 0.03; *p* < 0.001). However, the levels of secreted MCP-1, IL-6 and CXCL1 from mesangial cells challenged by IgAN-IgG-dd-IgA1 and HC-IgG-dd-IgA1 were comparable.

**Conclusions:**

We found that glycan-specific IgG antibodies derived from patients with IgAN had the biological effect to induce mesangial cells proliferation. Moreover, in the present study we also established a method for in vitro preparation of pathogenic IgG-ddIgA1 complexes, which could be applied in future studies exploring IgAN pathogenesis.

## Background

IgA nephropathy (IgAN) is the most common primary glomerulonephritis in the world, characterized by mesangial IgA deposits [1, 2]. IgAN is a highly heterogeneous disease, and patients with IgAN presented with highly variable clinical, pathological features and prognosis [3]. However, the pathogenesis of IgAN is not fully understood till today.

In recent years, accumulating evidences proved that circulating IgA1-containing immune complexes played important roles in the initiation and development of IgAN, because they could not only induce mesangial cells proliferation and matrix expansion, but also activate mesangial cells to secret multiple inflammatory factors, including IL-6, MCP-1, TGF-β and so on [4–6]. Moreover, the mesangial induced inflammatory factors could further damage other glomerular intrinsic cells through cross-talks among these cells, including mesangial cells, podocytes and tubular epithelial cells [7–11].

Although the exact composition of circulating IgA1-containing immune complexes in patients with IgAN are still unclear today, galactose-deficient IgA1 (Gd-IgA1) and glycan-specific IgG antibody have been identified as major two components. Moreover, in recent years, soluble CD89, secretory component and complement C3 were also identified as components in IgA1-containing immune complexes [12–14]. There has been a lot of studies revealed the importance of Gd-IgA1 in IgAN. Reports from multiple countries showed that, compared with healthy controls, patients with IgAN had higher levels of Gd-IgA1, which were also associated with disease progression [15–17]. Further studies revealed that heat aggregated galactose-deficient IgA1 molecules could induce mesangial cells activation, as well as neutrophils priming in vitro [18, 19]. Regarding glycan-specific IgG antibody, Suzuki et al. reported its elevated levels in patients with IgAN [20]. Moreover, the elevated levels of glycan-specific IgG antibody were found to be not only associated with urine protein excretion, but also with poor long-term renal outcomes [21]. Wada Y et al. also found that mesangial IgG deposition was associated with more severe clinical features in IgAN patients [22]. All these findings from observational studies focused on glycan-specific IgG antibody implied its pathogenic role in IgAN. However, we still lack direct evidences to clarify the biological effect of glycan-specific IgG antibody in IgAN.

In the present study, we in vitro prepared IgA1-containing immune complexes, using uniform aberrant glycosylated IgA1 molecules, and IgG from different individuals, to evaluate the injury effect of glycan-specific IgG antibody on mesangial cells.

## Methods

### Study population

In our present study, 35 patients with IgAN and 17 age and gender matched healthy controls were recruited. Diagnosis of IgAN was based upon the presence of dominant IgA deposition in mesangial area by immunofluorescence, and confirmed by light microscopy and electronic microscopy. Patients with Henoch-Schonlein purpura, liver cirrhosis and other secondary etiologies of IgAN were excluded by detailed clinical and laboratory examinations.

For recruited patients with IgAN, clinical features at the time of renal biopsy, including serum creatinine levels, 24-h urine protein excretion and hypertension, were collected from the medical records. Hypertension was defined as a systolic blood pressure (SBP) of 140 mmHg or more, or a diastolic blood pressure (DBP) of 90 mmHg or more, or taking antihypertensive medications to prevent high blood pressure. The glomerular filtration rate (GFR) of IgAN patients was calculated using the Modified Glomerular Filtration Rate Estimating Eq. [23]. Histologically, the Oxford classification was used for the evaluation of pathological lesions in biopsy specimens [24]. Additionally, crescent scores of C0 (no crescents), C1 (crescents in less than one fourth of glomeruli) and C2 (crescents in over one fourth of glomeruli), which were recently added to the Oxford classification, were also used in our present study [25, 26].

### Affinity chromatography

For each recruited individuals, 10 ml anti-coagulated (EDTA) peripheral venous blood was obtained. Then, plasma was isolated and frozen at −80 °C immediately pending isolation of IgA1 and IgG by affinity chromatography. At first, IgA1 fractions were purified from serum by agarose-bound jacalin affinity chromatography column (Pierce Chemical Company, Rockford, IL, USA), and then the flow-through were applied to protein G affinity column (Amersham Pharmacia) for isolation of IgG fractions. After eluted from agarose-bound jacalin, the IgA1 fractions were dialyzed against PBS, concentrated using Vivaspin (Sartorius Stedim Biotech, Sartorius, Goettingen, Germany) and separated by gel filtration chromatography using Sephcrl S300 to obtain monomeric IgA1. IgG fractions were eluted from protein G affinity column with acidic buffer (0.5 M acetic acid adjusted to pH 3.0 with ammonium hydroxide) and neutralized to pH 7.0 by 2 mol/l Tris–HCl, pH 9.0 immediately. Then, the IgG fractions were also dialyzed against PBS and concentrated using Vivaspin.

### Preparation of deglycosylated IgA1 and IgG-ddIgA1 complex

The purified monomeric IgA1 fractions from the recruited 17 healthy individuals were pooled and digested with neuraminidase from *Clostridium perfringens* (Sigma, St Louis, MO USA), and h-galactosidase from bovine testis (Sigma, St Louis, MO USA) to obtain de-sialic acid/de-galactose IgA1 molecules (ddIgA1) as described before [27]. In brief, 1 mg monomeric IgA1 were dissolved in 0.2 M sodium acetate buffer (pH 5.0), and added with 0.08 U neuraminidase and 0.02 U h-galactosidase to remove both sialic acid and Galactose. After incubation at 37 °C for 18 h, the ddIgA1 were dialyzed against PBS and concentrated using Vivaspin.

For in-vitro preparation of IgG-deglycosylated IgA1 complexes, a mixture of ddIgA1 and IgG (1:5 mass ratio) were incubated at 4 °C for 48 h, and then separated by gel filtration chromatography using Sephcrl S300 to obtain IgG-deglycosylated IgA1 complexes. The IgG-deglycosylated IgA1 complexes were also dialyzed against PBS and concentrated for cell culture experiments.

Meanwhile, ddIgA1 were separated by gel filtration chromatography using Sephcrl S300 to obtain monomeric ddIgA1 (mddIgA1) and polymeric ddIgA1 (pddIgA1), both of which were used as controls to treat cultured mesangial cells.

### Detection of glycosylation status of IgA1 and ddIgA1

For the detection of glcosylation status of IgA1 and deglycosylated IgA1, pooled monomeric IgA1 derived from healthy controls and ddIgA1 (1 μg/mL) were coated onto MaxiSorp plastic plates (Nalge-Nunc, Rochester, NY) at 4 °C overnight. After being blocked with 1% bovine serum albumin (Sigma, St Louis, MO USA), biotin-labeled SNA (1:500 dilution; Sigma) and biotin-labeled VVL (1:200 dilution; Sigma) were added and incubated for 1 h at 37 °C for the detection of sialic acid (SA) and N-Acetylgalactosamine (GalNAc). After washing, the plates were further incubated with horseradish peroxidase-ExtrAvidin (Sigma) for 1 h at 37 °C. The reaction was developed using 3,3′,5,5′-tetramethylbenzidine (TMB) liquid substrate system for ELISA (Sigma, St Louis, MO USA) and stopped with 1 mol/L sulfuric acid before the absorbance was measured at 450/570 nm.

### Western blotting

In vitro prepared IgG-ddIgA1 complexes (0.2μg) were separated by SDS-polyacrylamide gel electrophoresis (SDS-PAGE) and transferred onto a polyvinylidene difluoride (*PVDF*) membrane. After blocking, the membrane was incubated with HRP-conjugated mouse anti-human IgA antibody (AbD Serotec, Kidlington, UK) and HRP-conjugated rabbit anti-human IgG antibody (Abcam, Cambridge, UK) at 4 °C overnight. Binding was detected by western lightning plus ECL reagent (PerkinElmer, Waltham, MA, USA).

### Mesangial cell culture and treatment

Primary human renal mesangial cells were purchased from ScienCell™ Corporation (ScienCell™, Carlsbad, CA, USA) and maintained in mesangial cell medium (MCM) supplemented with mesangial cell growth supplement (MsCGS), 5% fetal bovine serum (FBS), penicillin G (100 U/ml) and streptomycin (100 U/ml) at 37 °C in a humidified 5% CO_2_ incubator according to the manufacturer’s specifications. After serum starving for 18 h, HMCs were treated with 100 μg/ml in vitro prepared IgG-dd-IgA1 complexes for 48 h. Meanwhile, 100 μg/ml mddIgA1, pddIgA1, IgG from healthy control (HC-IgG) and IgG from IgAN patients (IgAN-IgG) were used as controls to treat HMCs, respectively. After centrifugation, supernatants of cultured mesangial cells were collected and stored at −80 °C until the detection of inflammatory cytokines. Proliferation of cultured human mesangial cells were detected by MTS method using the CellTiter 96® AQueous One Solution Cell Proliferation Assay (Promega, Madison, WI, USA) according to the manufacturer’s instructions.

### Detection of inflammatory cytokines derived from cultured mesangial cells

For the detection of IL-6, MCP-1 and CXCL1 derived from cultured human mesangial cells, supernatants of mesangial cells treated with 100 μg/ml IgG-dd-IgA1 complexes for 48 h were used. Standard sandwich ELISA assays using DuoSet human ELISA kits (R&D Systems, Minneapolis, MN, USA) were performed for the detection according to the manufacturer’s specifications.

### Statistical analyses

Statistical analyses were performed by SPSS software (version 18.0; SPSS, Chicago, IL, USA). For data description, normally distributed quantitative variables were expressed as mean ± standard deviation. For non-normally distributed variables, median and interquartile range (IQR) were used. Categorical data were summarized as absolute frequencies and percentages. For Continuous variables, independent-samples t-test was used if the data was normally distributed, and if not, Mann–Whitney tests were performed. A two-tailed *p*-value less than 0.05 was considered statistically significant.

## Results

### Verification of successful preparation of ddIgA1 and IgG-ddIgA1 complexes

**Fig. 1:**
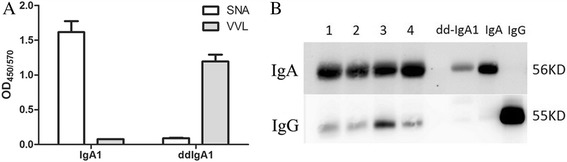
Verification of successful preparation of ddIgA1 and IgG-dd-IgA1 complexes. **a** Glycosylation status of IgA1 and ddIgA1 were detected by two lectins, SNA and VVL, which recognized sialic acid (SA), and N-Acetylgalactosamine (GalNAc), respectively. IgA1 molecules showed high binding capacity to SNA (blank column) and low binding capacity to VVL (filled column). In contrary, ddIgA1 showed low binding capacity to SNA (blank column) and high binding capacity to VVL (filled column). **b** Existence of dd-IgA1 (upper panel) and IgG (lower panel) in IgG-ddIgA1 complexes were tested by Western blot analysis. Both IgA1 and IgG were existence in IgG-ddIgA1 complexes of recruited individuals (lane 1–4). Isolated ddIgA1 (lane 5), IgA1 (lane 6) and IgG (lane 7) were used as references

We used lectin based ELISA to detect the glycosylation status of in vitro prepared ddIgA1 molecules. Two lectins, SNA, which recognized sialic acid (SA), and VVL, which recognized N-Acetylgalactosamine (GalNAc) were used. Compared with IgA1 molecules, ddIgA1 showed less binding to SNA and more binding to VVL (Fig. 1A), which indicated that ddIgA1 presented with decreased sialic acid and increased exposure of GalNAc, resulted by galactose deficient.

Western blot analysis was used to verify the existence of dd-IgA1 and IgG in IgG-ddIgA1 complexes. We detected both IgA1 and IgG in IgG-ddIgA1 complexes (Fig. 1B), implied successful in vitro preparation of the complexes.

### IgG-ddIgA1 complexes derived from IgAN patients induced activation of human mesangial cells

**Fig. 2:**
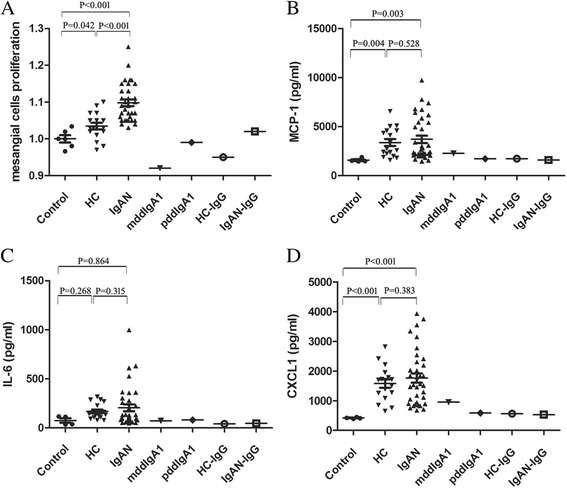
The biological effect of IgG-ddIgA1 complexes on cultured human mesangial cells. IgG-ddIgA1 complexes from both patients with IgA nephropathy (IgAN-IgG-ddIgA1) and healthy controls (IgAN-IgG-ddIgA1) could induce mesangial cells proliferation (**a**) and up-regulated excretion of MCP-1 (**b**), IL-6 (**c**) and CXCL1 (**d**). The levels of mesangial cells proliferation induced by IgAN-IgG-dd-IgA1 were significantly higher than those induced by HC-IgG-dd-IgA1 (1.10 ± 0.05 vs. 1.03 ± 0.03; *p* < 0.001), while the levels of MCP-1, IL-6 and CXCL1 derived from human mesangial cells when treated by IgAN-IgG-ddIgA1 and HC-IgG-ddIgA1 were comparable (MCP-1: 3701.1 ± 2199.9 pg/ml vs. 3373.9 ± 1465.6 pg/ml, *p* = 0.528; IL-6: 103.2 (81.5–277.4) pg/ml vs. 143.9 (108.2–248.6) pg/ml, *p* = 0.315; CXCL1: 1762.3 ± 934.0 pg/ml vs. 1575.8 ± 582.3 pg/ml; *p* = 0.383). In addition, monomeric ddIgA1 (mddIgA1) and polymeric ddIgA1 (pddIgA1), IgG derived from healthy controls (HC-IgG) and IgG derived from patients with IgAN (IgAN-IgG) were used as controls

IgG-ddIgA1 complexes from both patients with IgA nephropathy (IgAN-IgG-ddIgA1) and healthy controls (HC-IgG-ddIgA1) could induce the proliferation of mesangial cells and up-regulate the expression of multiple inflammatory cytokines, including IL-6, MCP-1 and CXCL1. Moreover, the levels of mesangial cells proliferation induced by IgAN-IgG-ddIgA1 were significantly higher than those caused by HC-IgG-ddIgA1 (1.10 ± 0.05 vs. 1.03 ± 0.03; *p* < 0.001, Fig. 2A). However, the levels of IL-6, MCP-1 and CXCL1 derived from human mesangial cells when treated by IgAN-IgG-ddIgA1 and HC-IgG-ddIgA1 were comparable (MCP-1: 3701.1 ± 2199.9 pg/ml vs. 3373.9 ± 1465.6 pg/ml, *p* = 0.528, Fig. 2B; IL-6: 103.2 (81.5–277.4) pg/ml vs. 143.9 (108.2–248.6) pg/ml, *p* = 0.315, Fig. 2C; CXCL1: 1762.3 ± 934.0 pg/ml vs. 1575.8 ± 582.3 pg/ml; *p* = 0.383, Fig. 2D). On the other side, mddIgA1, pddIgA1, HC-IgG and IgAN-IgG showed little biological effects in inducing HMCs proliferation and secretion of inflammatory factors, including IL-6, MCP-1 and CXCL1, which suggested that IgG, especially those from IgAN patients, had a synergic role to IgA1, when form the complex, to induce the activation of human mesangial cells.

## Discussion

IgAN is a primary glomerulonephritis characterized by deposition of IgA in glomerular mesangial area. Mesangial cells proliferation and inflammatory cells infiltration are the most popular histological lesions in patients with IgAN. Additionally, multiple inflammatory factors, including IL-6, IL-8, CXCL1, TGF-β1, MCP-1 and TNF-α, are up-regulated in the urine samples of IgAN patients [10]. Thus, to evaluate the kidney injury effect in IgAN using in vitro mesangial cells model, mesangial cells proliferation and inflammatory factor levels were widely used indices. In this study, we detected mesangial cells derived MCP-1, IL-6 and CXCL1 levels, as well as mesangial cells proliferation, to estimate the pathologenic effect of IgG-ddIgA1 complexes, mainly focused on anti-glycan IgG antibody.

Our results showed that in vitro prepared IgG-ddIgA1 complexes could not only induce proliferation of mesangial cells, but also up-regulate the secretion of MCP-1, IL-6 and CXCL1 from mesangial cells, which implied that our in vitro prepared IgG-dd-IgA1 complexes could induce mesangial cells activation, similar as circulating IgA1-containing immune complexes isolated from patients with IgAN [4, 5]. Actually, circulating IgA1-containing immune complexes were widely used in studies for exploring IgAN pathogenesis using in vitro mesangial cells model [9, 10]. However, the content of IgA1-containing immune complex in circulation was limited, thus, restricted its wide application in many studies. In the present study, we established a method for in vitro IgG-dd-IgA1 complexes preparation, using monomeric IgA1 and IgG, both of which were abundant proteins in circulation. Therefore, our method here would facilitate researchers to get large amount of pathogenic IgA1-IgG complex for future in vitro studies on IgAN pathogenesis.

Many previous studies proved the biological renal injury effect of circulating IgA1-containing immune complexes derived from IgAN patients, which contained both galactose-deficient IgA1 and glycan-specific IgG antibody [4, 7, 10]. Since aberrant glycosylated IgA1 molecules were identified to could induce mesangial cells activation, and the glycosylation levels of IgA1 molecules in the immune complexes from different individuals showed a large variation, it is hard to evaluate the independent biological effect of glycan-specific IgG antibodies using circulating IgA1-containing immune complexes. To overcome this problem, we used uniformly prepared ddIgA1 molecules to bind with IgG from different individuals for the IgG-ddIgA1 complexes preparation, in order to compare the biological effect of glycan-specific IgG antibodies between IgAN patients and healthy controls. We found that the levels of mesangial cells proliferation caused by IgG-ddIgA1 derived from IgAN patients were significantly higher than those from healthy controls, serving a reminder that glycan-specific IgG antibodies from IgAN patients could induce the proliferation of mesangial cells. However, the levels of inflammatory factors secreted from mesangial cells challenged by IgG-ddIgA1 from IgAN patients and healthy controls were comparable. Both kinds of IgG-ddIgA1 complexes, no matter derived from IgAN group or healthy control group, could similarly up-regulate inflammatory factors expression, which suggested this inflammatory effect should not be attributed to glycan-specific IgG antibodies, but might by induced by the uniform ddIgA1 in the complexes. Previously, Amore et al. reported that ddIgA1 could activate complement system and induce inflammatory response, which in according with our present findings [28].

## Conclusions

We found that glycan-specific IgG antibodies derived from patients with IgAN had the biological effect to induce mesangial cells proliferation. Moreover, in the present study we also established a method for in vitro preparation of pathogenic IgG-ddIgA1 complexes, which could be applied in future studies exploring IgAN pathogenesis.

## Abbreviations

cIgA1: Circulating IgA1-containing immune complexes
DBP: Diastolic blood pressure
GalNAc: N-Acetylgalactosamine
Gd-IgA1: Galactose-deficient IgA1
GFR: glomerular filtration rate
HC-IgG-dd-IgA1: IgG-ddIgA1 complexes from healthy controls
IgAN: IgA nephropathy
IgAN-IgG-dd-IgA1: IgG-ddIgA1 complexes from patients with IgA nephropathy
SA: Sialic acid
SBP: Systolic blood pressure

## References

[1] D'Amico G (1987). The commonest glomerulonephritis in the world: IgA nephropathy. Q J Med.

[2] Donadio JV, Grande JP (2002). IgA nephropathy. N Engl J Med.

[3] Schena FP (1990). A retrospective analysis of the natural history of primary IgA nephropathy worldwide. Am J Med.

[4] Novak J, Tomana M, Matousovic K (2005). IgA1-containing immune complexes in IgA nephropathy differentially affect proliferation of mesangial cells. Kidney Int.

[5] Novak J, Raskova KL, Suzuki H (2011). IgA1 immune complexes from pediatric patients with IgA nephropathy activate cultured human mesangial cells. Nephrol Dial Transplant.

[6] Tsuge T, Suzuki Y, Shimokawa T (2003). Monocyte chemoattractant protein (MCP)-1 production via functionally reconstituted Fcalpha receptor (CD89) on glomerular mesangial cells. Inflamm Res.

[7] Chan LY, Leung JC, Tsang AW, Tang SC, Lai KN (2005). Activation of tubular epithelial cells by mesangial-derived TNF-alpha: glomerulotubular communication in IgA nephropathy. Kidney Int.

[8] Lai KN, Leung JC, Chan LY (2008). Activation of podocytes by mesangial-derived TNF-alpha: glomerulo-podocytic communication in IgA nephropathy. Am J Physiol Renal Physiol.

[9] Lai KN, Leung JC, Chan LY (2009). Podocyte injury induced by mesangial-derived cytokines in IgA nephropathy. Nephrol Dial Transplant.

[10] Zhu L, Zhang Q, Shi S (2013). Synergistic effect of mesangial cell-induced CXCL1 and TGF-beta1 in promoting podocyte loss in IgA nephropathy. PLoS One.

[11] Lai KN, Tang SC, Leung JC (2011). Recent advances in IgA nephropathy--the glomerulopodocytic-tubular communication. Adv Otorhinolaryngol.

[12] Vuong MT, Hahn-Zoric M, Lundberg S (2010). Association of soluble CD89 levels with disease progression but not susceptibility in IgA nephropathy. Kidney Int.

[13] Zhang JJ, Xu LX, Liu G, Zhao MH, Wang HY (2008). The level of serum secretory IgA of patients with IgA nephropathy is elevated and associated with pathological phenotypes. Nephrol Dial Transplant.

[14] van Es LA, van den Wall Bake AW, Valentijn RM, Daha MR (1988). Composition of IgA-containing circulating immune complexes in IgA nephropathy. Am J Kidney Dis.

[15] Zhao N, Hou P, Lv J (2012). The level of galactose-deficient IgA1 in the sera of patients with IgA nephropathy is associated with disease progression. Kidney Int.

[16] Camilla R, Suzuki H, Daprà V (2011). Oxidative stress and galactose-deficient IgA1 as markers of progression in IgA nephropathy. Clin J Am Soc Nephrol.

[17] Suzuki H, Moldoveanu Z, Hall S (2008). IgA1-secreting cell lines from patients with IgA nephropathy produce aberrantly glycosylated IgA1. J Clin Invest.

[18] Diven SC, Caflisch CR, Hammond DK (1998). IgA induced activation of human mesangial cells: independent of FcalphaR1 (CD 89). Kidney Int.

[19] Lai KN, Leung JC (1993). Heat-aggregated IgA prepared from patients with IgA nephropathy increases calcium mobilization and superoxide production of human neutrophils in vitro. Nephron.

[20] Suzuki H, Fan R, Zhang Z (2009). Aberrantly glycosylated IgA1 in IgA nephropathy patients is recognized by IgG antibodies with restricted heterogeneity. J Clin Invest.

[21] Berthoux F, Suzuki H, Thibaudin L (2012). Autoantibodies targeting galactose-deficient IgA1 associate with progression of IgA nephropathy. J Am Soc Nephrol.

[22] Wada Y, Ogata H, Takeshige Y (2013). Clinical significance of IgG deposition in the glomerular mesangial area in patients with IgA nephropathy. Clin Exp Nephrol.

[23] Levey AS, Stevens LA, Schmid CH (2009). A new equation to estimate glomerular filtration rate. Ann Intern Med.

[24] Working Group of the International IgA Nephropathy Network and the Renal Pathology Society, Roberts IS, Cook HT, et al. The Oxford classification of IgA nephropathy: pathology definitions, correlations, and reproducibility. *Kidney. Int.* 2009;**76**:546–556. 10.1038/ki.2009.168 19571790

[25] Trimarchi H, Barratt J, Cattran DC, Cook HT, Coppo R, Haas M, Liu ZH, Roberts IS, Yuzawa Y, Zhang H, Feehally J (2017). IgAN Classification Working Group of the International IgA Nephropathy Network and the Renal Pathology Society; Conference Participants. Oxford Classification of IgA nephropathy 2016: an update from the IgA Nephropathy Classification Working Group. Kidney Int.

[26] Haas M, Verhave JC, Liu ZH (2017). A Multicenter Study of the Predictive Value of Crescents in IgA Nephropathy. J Am Soc Nephrol.

[27] Zhang JJ, Xu LX, Zhang Y, Zhao MH (2006). Binding capacity of in vitro deglycosylated IgA1 to human mesangial cells. Clin Immunol.

[28] Amore A, Coppo R (2000). Modulation of mesangial cell reactivity by aberrantly glycosylated IgA. Nephron.

